# WhatsApp-Based Record-Keeping System in a Private Neurosurgical Clinic Chain

**DOI:** 10.7759/cureus.45823

**Published:** 2023-09-23

**Authors:** Yousef M Odeibat, Mohammad Y Hiasat, Bilal Ibrahim, Waleed F Dabbas, Mohammad H Alhazaimeh, Qais A Samara, Ala Marji, Amer A Alomari

**Affiliations:** 1 Department of Neurological Surgery, Neuron Clinics, Amman, JOR; 2 Department of Neurological Surgery, Al-Balqa Applied University, Al-Salt, JOR; 3 Division of Neurosurgery, Department of Special Surgery, Faculty of Medicine, Al-Balqa Applied University, Al-Salt, JOR; 4 Department of Neurosurgery, Faculty of Medicine, Yarmouk University, Irbid, JOR; 5 Department of Neurosurgery, King Hussein Cancer Center, Amman, JOR; 6 Department of Neurosurgery, San Filippo Neri Hospital/Azienda Sanitaria Locale (ASL) Roma 1, Rome, ITA

**Keywords:** electronic health record system, neurosurgery, documentation, record-keeping, whatsapp

## Abstract

Background

The demanding nature of neurosurgical practice requires a reliable system for documentation and record-keeping. The cost of electronic health record systems can limit their availability in low- and middle-income countries. That is why less expensive and easily accessible technological alternatives should be sought. In this article, we describe our adopted system for medical record-keeping based on WhatsApp (Meta Platforms, Inc., Menlo Park, CA).

Methods

In our chain of six clinics, each clinic has its record-keeping WhatsApp group dedicated to sharing medical data of outpatients following up in that specific clinic and of inpatients cared for in hospitals in its area. After each encounter, our surgeons share smartphone-captured pictures of their patients' medical data on the WhatsApp group of the related clinic. The medical data are then categorized and stored by the secretary on the clinic’s computer to be accessed at any time for record retrieval.

Discussion

Our five years of experience with the WhatsApp-based record-keeping system with medical records of 11,729 patients proved to be reliable, cost-effective, user-friendly, and efficient, and it positively impacted patient care. Responsible behavior, security precautions, and regulating policies are essential to protect patient confidentiality.

Conclusion

Our system can be an inexpensive alternative to the electronic health record system in small healthcare facilities. It can help physicians practicing in low- and middle-income countries to improve medical records documentation, thereby improving patient care. There is a need for policies to regulate the use of instant messaging applications in professional medical communication.

## Introduction

The demanding nature of neurosurgical practice requires a reliable system for documentation and record-keeping. The cost of electronic health record (EHR) systems can limit their availability in low- and middle-income countries. That is why less expensive and easily accessible technological alternatives should be sought.

The importance of record-keeping in neurosurgery stems from the necessity to follow the progress of the neurological status of patients, follow the progress of radiological images, and for medicolegal concerns.

Practicing in a private clinic chain with clinics at different distant locations can complicate keeping track of patients' records, especially when most hospitals in developing countries are resource-limited and still use a paper-based record system. Furthermore, if the hospitals have an EHR system, it is intra-institutional and cannot be accessed from outside the hospital.

After the COVID-19 pandemic, the medical community was pushed to rely more on non-traditional communication technologies [1,2]. WhatsApp (Meta Platforms, Inc., Menlo Park, CA) is a cross-platform, free-of-charge, and user-friendly instant messaging application (IMA). Its features of flexible options to share text and media and the ability to make chat groups make it a convenient option to be used in medical practice [3].

In this article, we describe our WhatsApp-based record-keeping system adopted successfully and effectively for the past five years at our chain of six clinics distributed over the Hashemite Kingdom of Jordan. Then we analyze the advantages and concerns of this system.

## Materials and methods

The flow of our system is divided into record-keeping of inpatients, record-keeping of outpatients, categorization and storage of medical data, and retrieval of medical data.

Our chain of six clinics provides its services in four major cities in the Hashemite Kingdom of Jordan: two cities have one clinic each, and the other two cities have two clinics each. Each clinic is a separate storage station; in other words, the system is applied separately in each of our clinics.

Each clinic has its record-keeping WhatsApp group, dedicated to sharing medical data of outpatients following up in that specific clinic and of inpatients cared for in hospitals in its area. After each encounter, our surgeons share real-time smartphone-captured pictures of their patients' medical data on the WhatsApp group of the related clinic. Data shared on the WhatsApp groups include medical notes, radiological findings, and clinical observations from physical examination and laboratory results.

The clinic's secretary is a pivotal participant in these groups; the secretary's duty is to meticulously categorize and store the shared medical data on the clinic's computer to ensure easy accessibility of these data later on for various purposes such as patient care or research and analysis.

## Results

Upon encounter with an inpatient, pictures of the paper-based medical notes are captured by our surgeons using their smartphones. Also, pictures are captured of significant radiological images, reports (radiology, laboratory, histopathology, neurophysiology, etc.), clinical findings, and intraoperative findings. Pictures are then edited to remove unnecessary margins using basic photo editing features available on any smartphone.

**Figure 1:**
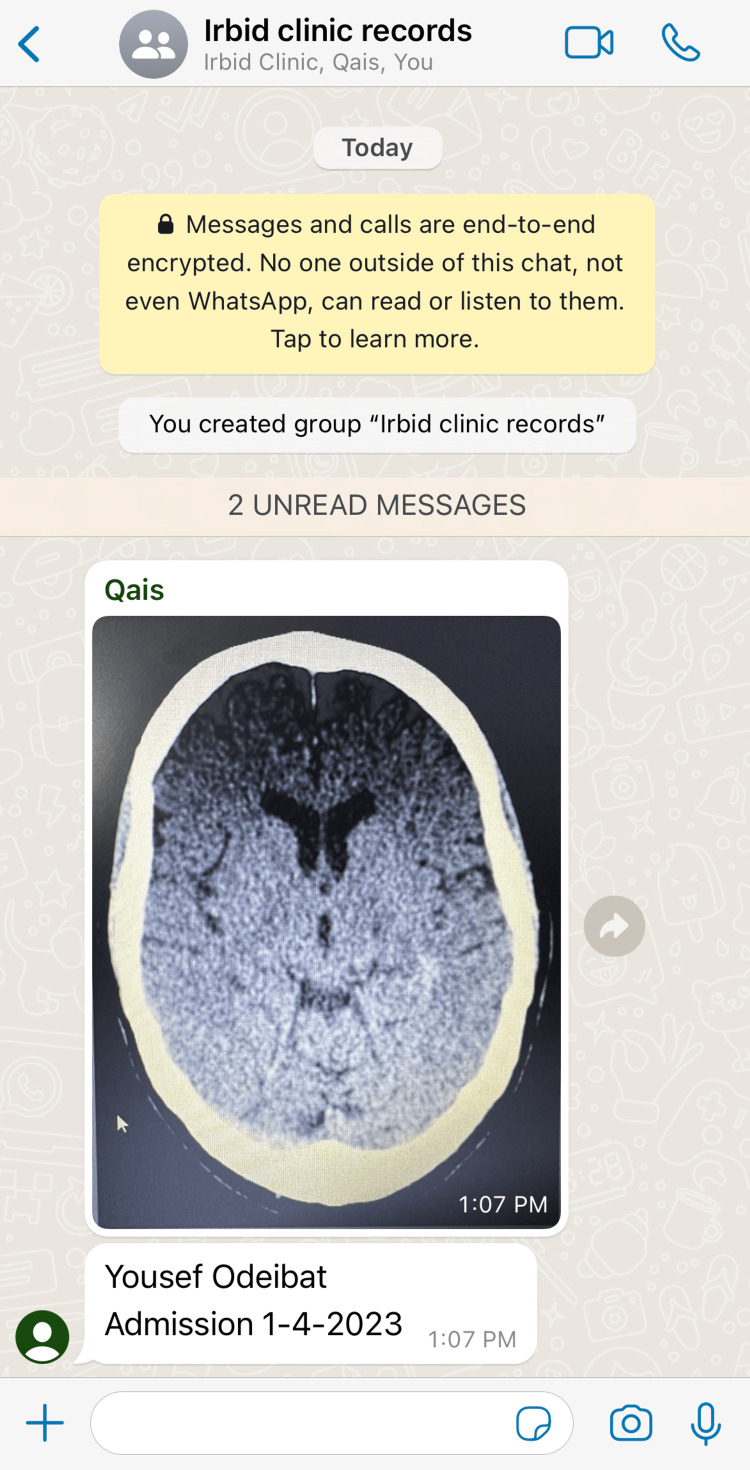
An illustrative example of medical data shared on a record-keeping WhatsApp group. A brain CT shared to "Irbid clinic records" WhatsApp group. The picture was captured by the treating surgeon, who evaluated the patient and examined the CT on the picture archiving and communication system (PACS). A message with the patient's name and the encounter's type and date is sent to the group to help in the later categorization of the medical records. Note WhatsApp's disclaimer, "Messages and calls are end-to-end encrypted. No one outside of this chat, not even WhatsApp, can read or listen to them." Yousef Odeibat is one of the authors of this article, and his name is used for illustrative purposes. Brain CT reproduced from File:001 Arteriovenous Malformation CT axial 01.png - Wikimedia Commons. Accessed: April 7, 2023.

These pictures are then shared on the record-keeping WhatsApp group of the related clinic. To facilitate later categorization of the data, the shared pictures are followed by a message with the patient's name and the type and date of the encounter (Figure 1).

**Figure 2:**
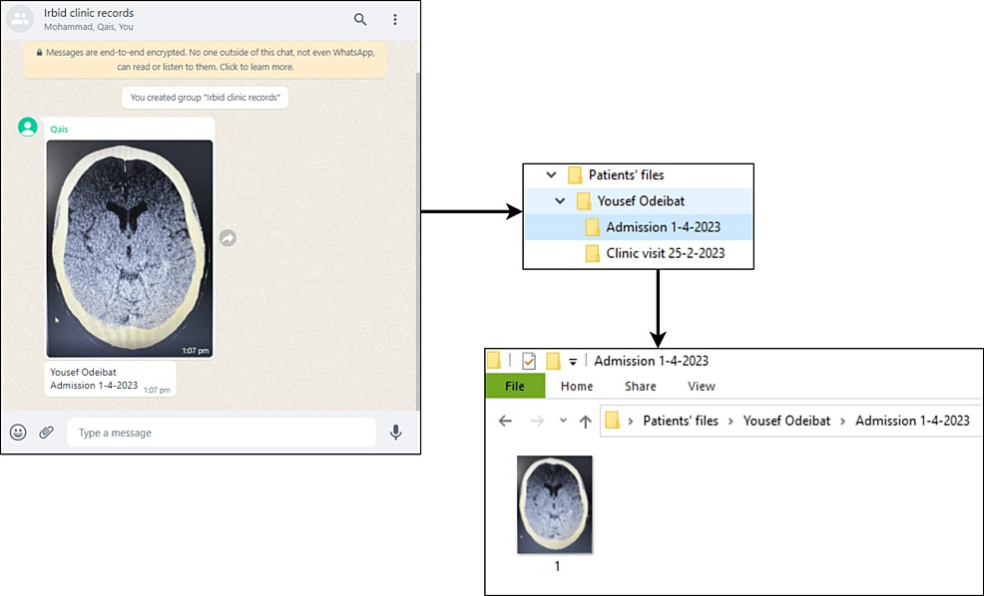
An illustrative example of categorization and storage of medical data on the clinic’s computer. Using the WhatsApp desktop application, the shared pictures of medical data are downloaded, categorized, and stored in a folder with the patient’s name “Yousef Odeibat” and a subfolder of the encounter’s type and date “Admission 1-4-2023.” Note that this patient has a previous encounter in the clinic; medical data from that encounter can be found in the subfolder “Clinic visit 25-2-2023.” Medical data are categorized in a hierarchical structure of nested folders and subfolders. Yousef Odeibat is one of the authors of this article, and his name is used for illustrative purposes. Brain CT reproduced from File:001 Arteriovenous Malformation CT axial 01.png - Wikimedia Commons. Accessed: April 7, 2023.

Using the WhatsApp desktop application, the shared pictures are downloaded to the clinic's computer by the secretary. Then the medical data are categorized and stored in a folder with the patient's name and a subfolder for that specific encounter (Figure 2).

For outpatients, clinic medical notes are written on a standardized paper form. Then the secretary scans and digitizes them on the clinic's computer. Also, smartphone pictures of significant radiological images, reports (radiology, laboratory, histopathology, neurophysiology, etc.), and clinical findings are captured. Our surgeons share these pictures on the record-keeping WhatsApp group of the related clinic. Pictures are followed by a message with the patient's name and the type and date of the encounter. Using the WhatsApp desktop application, the shared pictures are downloaded to the clinic's computer by the secretary. Then the scanned and smartphone-captured medical data are categorized and stored in a folder with the patient's name and a subfolder for that specific encounter.

The medical data are categorized and stored in a hierarchical structure of nested folders and subfolders on the clinic's computer, which the secretary operates. The “Patients' files” folder contains subfolders of all patients' names. In each patient-named subfolder, there is a text file named “Patient profile,” which contains the patient's name, sex, date of birth, and contact information. The patient-named subfolder also contains sub-subfolders for patient encounters' types and dates; these sub-subfolders contain pictures of medical records of that specific encounter for that specific patient (Figure 2). The types of encounters we use to name folders are admission, clinic visit, ER visit, consultation, or surgery. The “Patients' files” folder is manually copied using a portable external hard drive from the secretary's computer to update a similar folder on the doctor's office computer once weekly.

**Figure 3:**
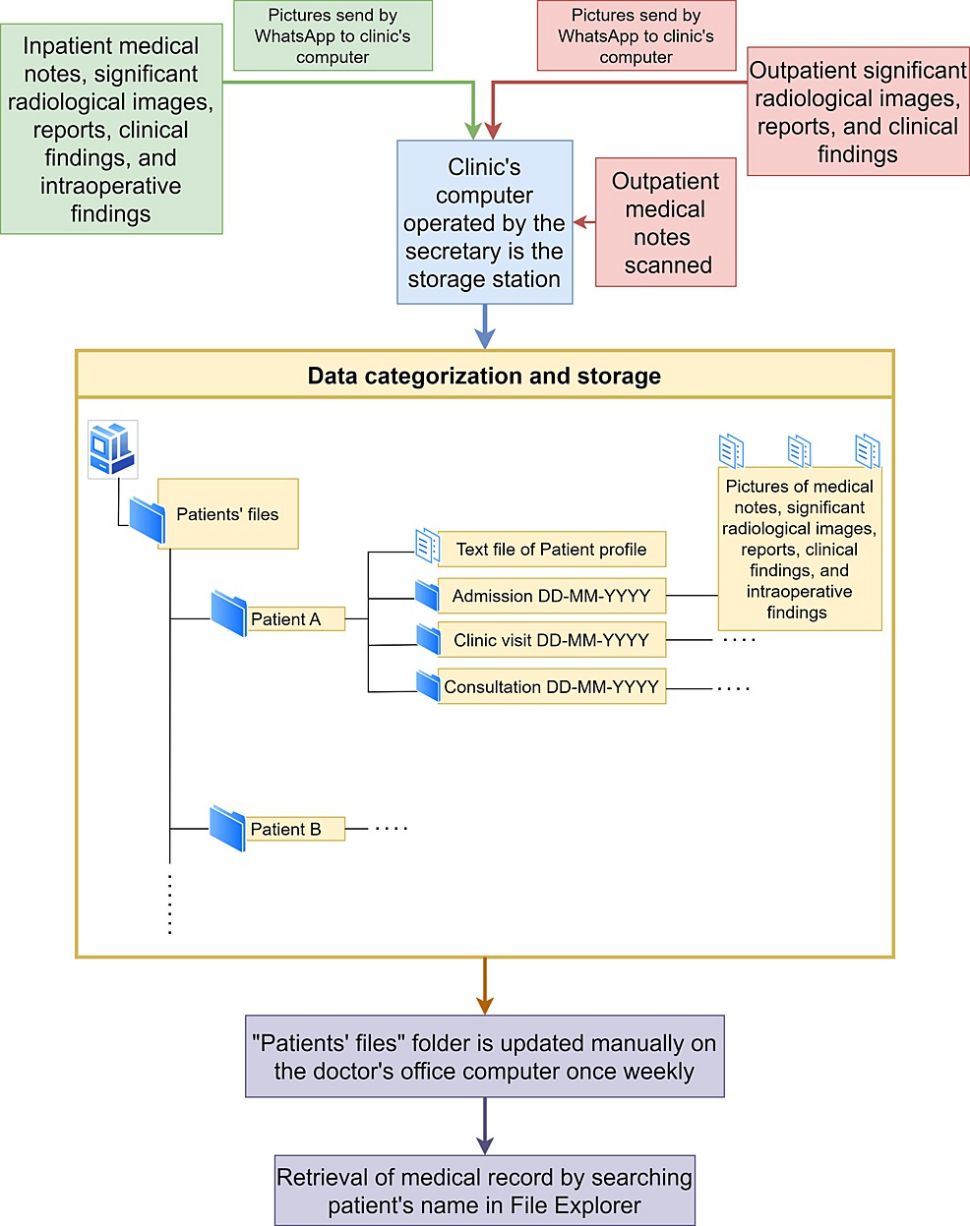
Flowchart of the WhatsApp-based record-keeping system.

To retrieve medical records, the “Patients' files” folder is accessed and searched for the desired patient's name using the search bar in File Explorer of Microsoft's Windows operating system (Microsoft Corporation, Redmond, WA). Figure 3 flowchart summarizes our WhatsApp-based record-keeping system.

## Discussion

WhatsApp can offer a cost-effective, user-friendly, and efficient communication option in neurosurgical practice [4,5]. It proved to be useful for professional communication in different medical disciplines such as ophthalmology [6], emergency medicine [7], radiology [8], dermatology [9], orthopedics [10], pathology [11], and burn care [12]. WhatsApp facilitates physician-physician communication [13], improves medical care [14], and enhances decision-making [15]. It also plays a significant role in physician-patient communications for remote monitoring and teleconsultation, especially in developing countries [16,17].

An international survey of neurosurgeons from 14 countries showed that 18 out of 22 responders believed that messaging service utilization improves patient care [18]. WhatsApp was the most used IMA in this survey, and 14 of the surveyed centers used chat groups to transmit patient-related medical data.

Our method of record-keeping is relatively inexpensive. The cost includes two computers for each clinic, internet service bills, the cost of smartphones, and monthly cellphone bills. WhatsApp is a free-of-charge application. The total cost of two computers in all clinics is approximately $12,687, and the yearly cost of internet service bills for all clinics is approximately $2,781. The average cost of six smartphones is $1,796 [19], with an estimated yearly cost of cellphone bills of $1,440. The total estimated cost is $18,704 in the first year and $4,221 yearly for internet and cellphone bills after that, compared to the estimated cost of an EHR system for a five-person practice, which can reach $162,000 in the first year and $85,000 a year in maintenance costs thereafter (Table 1) [20]. Bearing in mind that our computers, smartphones, internet, and cellphone bills are used for duties other than record-keeping. Our method can be a cost-effective alternative to the expensive EHR system in developing and resource-limited countries.

**Table 1:** Comparison between the estimated cost of establishing and maintaining the WhatsApp-based record-keeping system and the EHR system. EHR: electronic health record.

Cost of WhatsApp-based record-keeping system		Cost of EHR system	
Computers	2 computers x 6 clinics x $1,057.24, approximate cost of each computer ≈ $12,687	Establishing cost	$162,000
Phones	6 phones x $299.3, the average cost of a smartphone in 2022 ≈ $1,796		
Yearly internet service bill	6 clinics x 12 months x $38.62, monthly internet service bill ≈ $2,781		
Yearly cell phone service bill	6 phones x 12 months x $20, estimated monthly bill ≈ $1,440		
The total cost of the first year	$18,704		
Yearly maintenance cost	$4,221	Yearly maintenance cost	$85,000

Our system's ease makes it practical. It has been used efficiently for the past five years of our experience. It improved the communication between our surgeons and positively impacted patient care [21] and follow-up; using WhatsApp for record-keeping is just at our fingertips. In our experience, retrieval of these records assisted our decision-making, but it has not been studied systematically. Until October 2022, we have had medical records of 11,729 patients distributed over six clinics, with a total storage size of 29.1 gigabytes. If this amount of medical data was paper-based, the filing, storing, and retrieving of medical records would be time-consuming, and the infrastructure and human resources costs would be significant [22,23].

The reliability of smartphone-captured pictures of radiological images shared through IMAs compared to images viewed on picture archiving and communication systems (PACS) should be investigated more thoroughly. In Yamada's et al. [24] experience, one image slice selected and captured by a neurosurgeon's phone is sufficient to represent the diagnosis. Stahl et al. [25] concluded that video clips of CT scans of thoracolumbar fractures captured by a smartphone camera and shared on WhatsApp are reliable for diagnosing, classifying, and proposing treatment.

Kalra et al. [26] found that brain CT scans of acute stroke patients captured using a smartphone and shared on WhatsApp are perfectly reliable (κ = 1) in diagnosing intracerebral hemorrhage. However, diagnosing early infarct signs, stroke mimics, and proximal vessel occlusion was less reliable. Inan et al. [27] studied WhatsApp evaluation of brain CT in trauma cases and found perfect reliability (κ = 1) for intracerebral hemorrhage diagnosis but less reliability in identifying normal findings, subdural hematoma, epidural hematoma, and fractures. However, diagnosing subarachnoid hemorrhage and parenchymal contusion had the lowest reliability.

Joshi et al.'s [5] experience with the WhatsApp referral service suggests that it can be used effectively to refer neurosurgical patients and their images to a tertiary referral center. However, the most commonly missed diagnoses in transferred images include subacute subdural hematoma, small aneurysms, subdural empyema, and linear fractures. No adverse clinical events were solely related to the use of the WhatsApp referral service. Bullard et al. [28] also found that using WhatsApp to communicate CT scans significantly affects the transfer decisions of neurosurgical patients from referring hospitals to a level 1 trauma center. All the evidence suggests that record-keeping of radiological images captured by a smartphone can hold sufficient information to be considered a reliable tool for diagnosis and follow-up and thus to be part of patients' medical records.

In our system, we only take pictures, but not videos, of significant radiological findings. We intend to make our data brief but comprehensive and reduce the data storage size. It should be emphasized that smartphone-captured pictures should never be considered a substitute for PACS due to the reduced quality, compared to the naked eye, and the lack of whole radiological slices and sequences. The quality of the captured pictures can vary depending on the operator's photographic skills and the phone's hardware [5]. To overcome these limitations, we maximize the quality of pictures by using smartphones with high-quality cameras. Also, the treating surgeon selects and captures the image slices after reviewing the radiological images on PACS and choosing the best slices to represent the case to be attached to the medical record. We also record-keep radiological reports to decrease the likelihood of missing a radiological finding.

Our data are stored on our clinics' computers but not backed up to a server. If a computer malfunctions, its data may be lost forever. This is why the data are stored on two separate computers in each clinic, the secretary’s and the doctor's office computers. In addition, a portable external hard drive is used regularly to back up and transfer data between the two computers of each clinic. The lack of a data storage server limits the accessibility of the records to the specific clinic where the data are stored. However, this is usually not a problem, as our clinics are far from each other, and our patients usually continue follow-up in the same clinic. Using a cloud storage service such as Google Drive or Microsoft OneDrive would be an inexpensive accessibility improvement of our system.

Nevertheless, there are security and privacy concerns [29]. Our system uses the surgeons' personal smartphones, which can be a vulnerability in data security if a phone goes missing or the medical records are shared with the wrong contact. The Jordanian Medical and Health Liability Law protects patient confidentiality, but there is still no legislation to regulate the use of IMAs in medical practice in Jordan. The Health Insurance Portability and Accountability Act (HIPAA) [30] in the United States and the General Data Protection Regulation (GDPR) [31] in the European Union aim to protect data security; sharing identifiable health information on WhatsApp is incompatible with these regulations and is penalized by fine and imprisonment [32]. This is why our system cannot be applied in different settings due to legal issues.

WhatsApp is end-to-end encrypted [33], which means that encryption and decryption of messages sent and received on WhatsApp occur entirely on smartphones. According to WhatsApp company, this ensures that no one outside of the chat, not even WhatsApp, can read or listen to messages. The phones of our surgeons and secretaries are passcode-protected. Our computers are protected with firewalls and antivirus software that are regularly updated and maintained. Access to clinics' computers is limited to our surgeons and secretaries. In the misfortunate event, if a phone goes missing, proper proactive phone security settings can enable erasing phone data remotely. Moreover, our surgeons and staff's job contracts include a confidentiality agreement to protect patients' information. In our five years of experience with this system, we have not experienced any patient confidentiality breach or misuse.

It can be argued that patient confidentiality, even with conventional documentation systems, is not a utopia of privacy [34]. Every documentation system, whether paper-based, EHR, or WhatsApp-based, has unique security vulnerabilities. In any system, the security of patients' records can be compromised by healthcare providers who may view records of patients they are not involved in their treatment, or by staff members who may access patients' records for unauthorized purposes [35]. Also, both EHR [36] and WhatsApp-based systems can be a target for cyberattacks and hacking activities.

A balance should be made between the potential benefits and the security concerns [37] of the WhatsApp-based record-keeping system. A key point in any documentation system is to prevent records misuse and to commit to patients' trust that their health information is secure and protected. This is why responsible behavior, proper security precautions, and policies to regulate the use of IMAs in professional medical communication are essential to protect patient confidentiality and prevent medical data misuse.

Searching in our method is limited to patients' names. We cannot search for a specific diagnosis or a keyword. That makes data collection and analysis of our database difficult. To overcome this challenge, we have recently adopted a code list of the common diagnoses and surgical interventions encountered in neurosurgical practice. Patients' names and a code of their condition are entered into a database after each encounter; that database can ease later data collection.

## Conclusions

New communication technologies have made our lives easier and more practical. Our five years of experience with the WhatsApp-based record-keeping system with medical records of 11,729 patients proved to be reliable, cost-effective, user-friendly, and efficient, and it positively impacted patient care. Responsible behavior, proper security precautions, and policies to regulate the use of IMAs in professional medical communication are essential to protect patient confidentiality and prevent medical data misuse.

Our system can be an inexpensive alternative to the EHR system. It can help physicians practicing in low- and middle-income countries improve medical records documentation with a reasonable cost and effort, thereby improving patient care. Our system is best suited for small healthcare facilities. Settings not recommended to adopt our system are large hospitals with high patient flow and countries with regulations prohibiting WhatsApp use for sharing identifiable health information.
